# Cultural engagement predicts changes in cognitive function in older adults over a 10 year period: findings from the English Longitudinal Study of Ageing

**DOI:** 10.1038/s41598-018-28591-8

**Published:** 2018-07-05

**Authors:** Daisy Fancourt, Andrew Steptoe

**Affiliations:** Department of Behavioural Science and Health, University College London, 1-19 Torrington Place, London, WC1E 7HB UK

## Abstract

There is increasing evidence that leading an active, socially engaged lifestyle might protect against cognitive decline. The arts have been proposed as potentially beneficial activities due to their combination of cognitive complexity and mental creativity. Yet it remains uncertain which types of arts engagement and what level of engagement is required for potential benefits to accrue. This study therefore explored the association between three types of cultural engagement (visiting museums/galleries/exhibitions, going to the theatre/concert/opera and going to the cinema) and change in cognitive function over 10 years amongst adults aged over 52. Our participants (n = 3,445), drawn from the English Longitudinal Study of Ageing, were assessed in 2004/5 and 2014/15. We measured memory and semantic fluency at baseline and follow-up, analysing results using ordinary least squares regression models. Independent of demographic, health and social confounders, visiting museums/galleries/exhibitions and going to the theatre/concert/opera were associated with a lesser decline in cognitive function. Sensitivity analyses confirmed effects were unaffected by considerations of mobility or dementia diagnoses. However, going to the cinema was found to hold little effect for cognitive preservation. Overall, our results suggest that more frequent cultural engagement is associated with more marked effects, but even annual engagement may be protective.

## Introduction

Cognitive decline in older adults is a recognised public health challenge, with worsening memory and other cognitive abilities associated with a lack of functional independence and lower quality of life as well as signalling the onset of dementias^1–3^. As a result, identifying ways of delaying cognitive decline is recognised as a public health priority for aging societies^4,5^. One area of growing interest in epidemiological research has been whether spare-time leisure activities can protect against such cognitive decline; something that has been explored in a number of longitudinal studies. As examples, having a hobby, reading and engaging in cultural activities amongst people aged 41–88 are associated with increased intellectual functioning over a 20 year period^6^. Having a hobby is associated with reduced cognitive decline over the following 5 years in older adults aged 70–84^7^. Activities such as sewing, reading, playing music, playing cards, playing board games and attending the opera are associated with smaller declines in global cognition, language and executive function (but not episodic memory) in adults aged 65 and over^8^. And activities such as reading, painting, doing crosswords, gardening, cooking, sewing playing cards and cultural engagement are inversely associated with dementia incidence over the following 6.4 years in men and women aged 75 and over^9^. Consequently, systematic reviews of such studies have suggested that active and socially integrated lifestyles might be neuroprotective^10^.

However, most studies (including those cited above) have looked at ‘leisure’ broadly, with multiple different activities not studied individually but combined into indices. An important consideration is whether all of these different activities are equally beneficial or whether some have greater effects than others. While ‘leisure’ can be a broad term referring to any activities undertaken in one’s free time, existing studies have in particular highlighted the cognitive benefits of ‘complex’ and ‘creative’ tasks, as these activities are thought to build individuals’ capacity to deal with intellectual challenges and be linked with cognition^6,11,12^. Common to both ‘complex’ and ‘mental creative’ activities are activities involving the arts and culture. *Productive* cultural activities include singing in choirs, taking part in dance classes and engaging in crafts activities, while *receptive* cultural activities include going to the cinema, watching a concert or visiting a museum (also referred to as ‘cultural engagement’). Park *et al*. (2007) have proposed that it is primarily productive activities that have cognitive benefits^13^, suggesting that while receptive activities involve taking part in assimilatory behaviours that use existing skills and schema, productive activities require the acquisition of new skills and schema which accounts for their stronger cognitive effect^14^. To date, several studies have looked at productive cultural activities and cognition. For example, reading and playing musical instruments has been associated with better cognition as well as a lower risk of dementia and Alzheimer’s disease^15,16^. And comparisons of musicians and non-musicians have consistently demonstrated better preservation of cognitive function (including memory and executive function) in musicians across the lifespan^17,18^. However, apart from the inclusion of receptive cultural activities within broader leisure indices (as in the examples cited above), very little research has been undertaken on such engagement. Indeed, an fMRI study that compared 10-week programmes of art participation classes (‘productive’) vs art evaluation classes (‘receptive’) showed greater spatial improvement in functional connectivity (specifically between the posterior cingulate cortex and the frontal and parietal cortices) in the production rather than the reception group, questioning whether receptive cultural activities do confer cognitive benefits^19^. But a small number of other studies have described promising associations between receptive cultural engagement and cognition^20,21^. The challenge is that these have generally treated such activities as a single entity using a broad index, despite the fact that types of activities can be very different to one another. Comparing the effects of differing receptive cultural activities is important to understanding if and how receptive cultural activities are truly associated with preservation of cognitive function.

Consequently, the primary aim of this study was to explore the under-researched effects of three different types of receptive cultural activities on cognitive function amongst adults aged 50 and over across a 10 year period: going to an exhibition (such as an art gallery or museum), going to a live performance (such as the theatre, a concert or the opera) and going to a screen-based performance (such as the movies/cinema). The secondary aim was to consider what level of participation is required for cognitive benefit to accrue.

## Results

We assessed self-reports of three types of receptive cultural participation amongst 3,445 men (44.8%) and women (55.2%) aged 52 to over 90 years old (mean 62.9 years, SD = 7.5) and examined associations with cognition 10 years later. Our sample was drawn from the English Longitudinal Study of Ageing^22^; a nationally representative population cohort. A total of 69.4% reported visiting art galleries or museums, 73.9% reported going to the theatre, concerts or the opera and 68.4% of participants reported attending the cinema. Full participation profiles are shown in Table 1.

**Table 1:** Cultural participation.

	Gallery or museum	Theatre, concert or opera	Cinema
Never	1,055 (30.6%)	898 (26.1%)	1,088 (31.6%)
Less than once a year	954 (27.7%)	742 (21.5%)	845 (24.5%)
Once or twice a year	767 (22.3%)	873 (25.3%)	643 (18.7%)
Every few months	491 (14.3%)	662 (19.2%)	585 (17.0%)
Monthly or more	178 (5.2%)	270 (7.8%)	284 (8.2%)

Participation in cultural activities showed moderate inter-correlations, with going to galleries or museums correlating with going to the theatre, concert or opera (r = 0.57) and with going to the cinema (r = 0.49), while going to the theatre and going to the cinema also correlated (r = 0.55; all p < 0.001).

**Figure 1:**
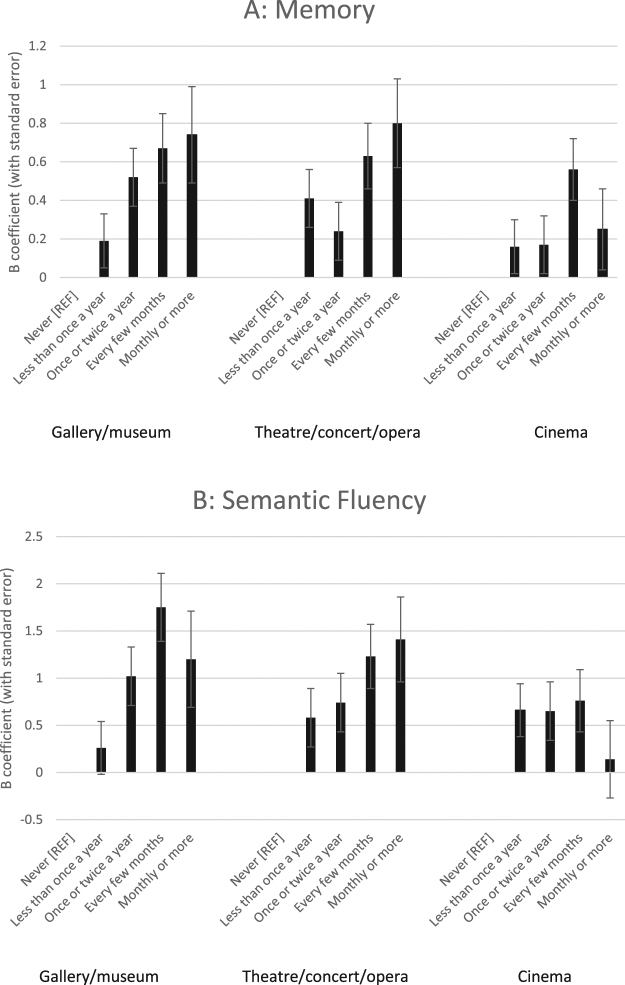
Associations between cultural engagement and (**A**) memory (**B**) semantic fluency.

We assessed cognition using a test of semantic fluency (which is often regarded as a measure of executive function since it involves self-initiated activity, organisation and abstraction, response inhibition and set shifting and calls upon memory and language processes) and an immediate and delayed recall task as a measure of memory; both measures that have been shown to be predictors of clinically significant cognitive decline^23,24^. Ordinary least squares regression was used to estimate the effects of each of the three types of cultural engagement on each of the two cognitive measures (semantic fluency and memory). Preliminary analysis models adjusted for baseline cognition, while further models additionally adjusted for factors that might influence cultural participation and cognitive function, including demographic variables (sex, age, marital status, ethnicity, educational attainment, employment status, occupational classification and wealth), health-related variables (including self-reported health, eyesight, hearing and depression) and social/activity-related variables (including social networks, civic and community engagement, whether participants had a hobby, whether participants used the internet and whether participants read a daily newspaper). While these different models showed that some of the associations between cultural engagement and cognition were partly explained by confounding variables, it is notable that neither socio-economic, health-related nor other social confounders led to an attenuation of associations between going to galleries/museums or going to the theatre/concert/opera. The B coefficients and standard errors for the fully-adjusted models for the association between cultural participation and cognitive function 10 years later are shown in Fig. 1, with full regression models being detailed in Tables S1D.

### Gallery/museum

Going to galleries and museums was associated with a smaller decline in cognitive function compared with non-participation. For memory any attendance was associated with lower cognitive decline, with a dose-response relationship indicating that more frequent attendance had a greater effect on cognition. For semantic fluency, attending once a year or more appeared to be protective These results all held when applying a Bonferroni correction for multiple comparisons, except the results for most frequent engagement in relation to semantic fluency.

### Theatre/concert/opera

Going to the theatre, concert or opera was also associated with a smaller decline in cognitive function. In relation to both memory and semantic fluency, attending once a year or more appeared to be protective with evidence of a dose-response relationship in particular for semantic fluency indicating that more frequent attendance had a greater effect on cognition. When correcting for multiple comparisons, results for attending every few months or more frequently remained significant.

### Cinema

Although going to the cinema was significantly associated with cognition when taking baseline cognitive function and demographic factors into account, results became inconsistent in the fully adjusted models. In relation to memory, attending every few months appeared to be protective, but there was no evidence that attending either more or less frequently than this had any benefits. In relation to semantic fluency, only attending on an infrequent basis appeared to have any protective effect and these results were attenuated when correcting for multiple comparisons.

We considered five further nested research questions. First, we considered cross-sectional associations between cultural participation and cognition at baseline in order to ascertain whether there were already cognitive differences evident when participants were first assessed. To do this, we reran the fully-adjusted longitudinal OLS regression model but substituted cognition at baseline as the dependent variable. There was no baseline association between going to concerts/theatre/opera and semantic fluency and little evidence of associations between going to the cinema and either semantic fluency or memory. However, there was an association between going to the concerts/theatre/opera and memory and going to museums/galleries and both memory and semantic fluency (shown in Supplementary Table S2). This confirmed the decision to include baseline cognition in all of the analyses to control for its effects.

Second, we explored whether age was a moderating factor by rerunning analyses including an interaction term showing cultural participation × age. There was no evidence of any significant moderating effects of age. Third, we explored whether effects on cognition were specifically found amongst those already showing signs of cognitive decline by stratifying the sample on the basis of baseline semantic fluency and memory (Supplementary Table S3). Although statistical power was reduced, results suggest that activities were protective across both those above and below the median level of cognition at baseline. Fourth, we considered that participants with mobility issues either involving walking or sitting might have had different patterns of engagement, so we excluded these participants and repeated our analyses (Supplementary Table S4). This actually increased the strength of associations, despite reducing the sample size by 16% (537 participants). Fifth, we considered that patterns of cultural engagement might be different amongst participants with dementia or on the cusp of developing dementia, so we excluded participants who reported a dementia diagnosis at baseline or in the two years following (n = 131) and repeated our analyses (Supplementary Table S5). This did not affect the significance of results.

## Discussion

It has been proposed that cultural engagement might be protective against cognitive decline. However, research has focused mainly on productive cultural engagement (involving ‘doing’ activities) and there has been less research into the effects of receptive cultural engagement. Furthermore, there has been little exploration of the differential benefits of different types of receptive cultural engagement nor what frequency of engagement is required for effects to be experienced. This study demonstrated that going to exhibitions (such as an art gallery or museum) and going to live performances (such as the theatre, a concert or the opera) could have benefits for both memory and semantic fluency amongst those of higher and lower cognitive status at baseline, independent of a range of potential demographic, health-related and activity-related variables. Sensitivity analyses showed that associations with memory were similar across the age spectrum. However, much less consistent results were found for screen-based performances (cinema attendance), with suggestions that viewing holds little benefit for semantic fluency or memory. Overall, significant results were unaffected by considerations of mobility or dementia diagnoses. Further, we gradually built up our regression models, which allowed us to see that, although socio-economic factors accounted for much of the association between cultural engagement and cognition, health-related factors such as depression did not account for much further association, and notably results were maintained even when controlling for other types of social activities, suggesting that it is not just that these cultural activities are proxies for wider social engagement, but the specific cultural component is important for cognition.

These results are correlational and do not demonstrate causal associations. Although we controlled statistically for a variety of sociodemographic, health, affective and social factors, it is possible that unidentified confounding factors are responsible for the effects observed. Nevertheless, a pertinent question is why visiting galleries and museums or going to concerts, theatre or the opera might have an effect on cognitive function, especially given that they are not inherently productive. There are a number of theories that could explain the effects found here. One potential explanation is cognitive reserve^25^. Complex and stimulating experiences can enhance neuronal structure and brain function through additional environmental stimulation and thereby provide a protective effect either against neurodegeneration or cognitive decline. This might be achieved through developing functionally more efficient cognitive networks even in the presence of morphological or functional deterioration of the brain^26,27^. This is one plausible explanation for the effects of galleries, museums, concerts, theatre and the opera. Another potential explanation is that these activities recruit bilateral neural circuits involved in cognitive activities. For example, studies of music have found that listening to polyphonic music specifically recruits bilateral temporal, frontal and parietal neural circuits that underly memory, attention, imagery and semantic and syntactic processing^28^. While galleries, museums and the theatre may not involve music per se, there may be similar cognitive mechanisms activated by these other cultural activities through their role in providing a stimulating environment.

Another explanation is the ‘use it or lose it’ hypothesis, which suggests that a lack of stimulation in everyday life can lead to faster deterioration in cognitive function (a ‘disuse syndrome’)^29^. Specifically, intellectually-stimulating activities (including those that involve novel information processing) have been associated with changes in cognitive functioning. Galleries, museums, concerts, theatre and the opera are all examples of intellectually-stimulating activities involving novel information so could still prevent cognitive ‘disuse’.

A third explanatory hypothesis concerns arousal and hedonic tone. Neuropsychological studies have demonstrated that positive affect leads to increased brain dopamine levels, which are associated with improved cognitive flexibility^30^. Enjoyable activities can induce positive affect and heighten arousal which has been shown to lead to improved cognitive performance^31^. Broader positive wellbeing is also linked with a reduced risk of cognitive decline amongst older adults at a population level^32^. There is a wide literature showing benefits for positive affect and wellbeing from engaging with cultural activities such as the theatre, concerts and museums^33^. A related explanation could also be that receptive cultural activities are stress-reducing; something that has been shown both psychologically and biologically^34^. Stress has been linked with faster cognitive decline, such as through weakened prefrontal networks, lower systolic blood pressure reactivity or increased cortisol levels^35–37^. So combined stress-reducing and affect-enhancing effects of receptive cultural activities provide another explanation for how receptive cultural activities could protect against cognitive decline.

A final explanation is that even receptive cultural activities that do not require active participation are inherently social in that they take place in locations with other people and involve social contact, even if one attends alone. McFadden and Basting (2010) have argued that the brain is a social organ and proposed that creative engagement may have a neuroprotective effect through supporting resilience^38^. They suggest that supportive, creative, accepting communities could specifically support the retention of cognitive capacity. Other studies have shown that social isolation and loneliness are associated with poorer cognitive function in older age^39^, whereas social activity can be protective against cognitive decline^40^. While these analyses controlled for wider social networks and social engagement in order to try and isolate the association between cultural participation and cognition, cultural engagement is a multimodal activity which includes a social component, and it remains possible that this social component of these cultural activities plays a role in the association with cognition. Consequently, there are a range of plausible theories relating to the environments created by receptive cultural activities, the mental stimulation they provide, their effects on stress and affect and their social nature that could explain the effects found here.

However, an equally important consideration is why there was a more mixed picture regarding the cognitive benefits from going to the cinema, with less consistent evidence that cinema attendance was associated with lower cognitive decline. One possibility is that going to the cinema has similar effects to television on cognition. Studies specifically on television and cognition have shown that television leads to a more alert but less focused brain, which could have implications for cognitive function^41^. Indeed, a study of adults aged 55 or over found that watching television was also associated with a higher risk of developing cognitive impairment over the following 5 years^42^. It is possible that cinema, as another screen-based activity, could have similar cognitive effects as television. Further, there are studies suggesting that screen-based activities such as watching television are associated with higher levels of mental health conditions such as depression^43^, which could lead to indirect effects on cognition. But this remains to be explored further.

Our cross-sectional sensitivity analyses are also revealing. The baseline associations between theatre/concert/opera and memory and museums/galleries and cognition generally suggest that past cultural engagement may already have affected cognition by the point of assessment for this study, or alternatively that those with better cognition across the lifetime were more likely to engage. But here, it is interesting to note that we found longitudinal associations over the following decade for those both with and without baseline indications of cognitive decline, suggesting that cultural engagement could have benefits both for those already experiencing cognitive impairment as well as those who show no such signs. In contrast, the lack of baseline correlation between going to the theatre/concert/opera and semantic fluency suggests either that longitudinal associations for this cultural activity were more specific to participation in older age or that adults engage more frequently in these activities as they get older, leading to later effects on cognition. In light of this, a key consideration is whether programmes to increase cultural engagement amongst older adults could specifically arrest a decline in cognitive function. Promising results have been found in studies of productive cultural activities involving older adults. Music programmes and combined photography and quilt-making classes have been found to improve executive function^44,45^, while theatre training has been found to improve memory^46^, and music-based multitask training has been found to reduce impairments in cognitive function^47^. However, these are all examples of productive cultural activities. Studies of receptive cultural engagement are limited, although there are some preliminary promising findings of visual art appraisal classes and memory^46^. Consequently, experimental studies on receptive cultural engagement programmes are encouraged.

This study had a number of methodological strengths. It involved a nationally representative sample of the population in England along with a relatively large sample size. In contrast to previous studies, it explored three specific types of receptive cultural engagement so was able to compare and contrast their effects. It also looked specifically at the frequency of attendance in relation to cognitive decline. However, our exploration of different types of receptive cultural engagement was limited to those measures included in the dataset. And we were unable, in this instance, to make a direct comparison of receptive and productive cultural activities. While we took account of all identified confounding variables, it is recognised that variables do not always adequately capture the latent construct being measured. Further, the findings should be interpreted in light of the study limitations. Causal conclusions cannot be drawn from observational studies. Nevertheless, baseline cognition along with a range of demographic, health-related and social/activity-related variables were included in analyses, ruling them out as being responsible for associations with cognition 10 years later. Our study also required participants to self-report their current engagement in cultural activities. While the question focused on engagement in the past year rather than over longer retrospective timescales, it is acknowledged that such reporting may still have been biased. Another limitation is that it is likely there is a bidirectional relationship between cultural and engagement and cognition, as has been shown with cognitive stimulation and cognitive status^6^. Consequently, future studies assessing the effect of cultural participation on cognition through specific interventions are needed. Future considerations could include whether specific receptive cultural activities are associated with reductions in the risk of dementia onset; a question that has been explored broadly in relation to leisure activities, but for which there remains a gap in the literature in relation to receptive cultural activities. Overall, this study suggests that pre-existing cultural assets such as galleries, museums, theatres, concerts and opera houses could play a role in supporting cognitive function in older adults. It suggests that more frequent cultural activity is associated with more marked effects, but even annual engagement may be protective.

## Methods

### Participants

This study involved the analysis of data from the English Longitudinal Study of Aging (ELSA); a longitudinal panel study of adults aged 50 or over living in England (www.elsa-project.ac.uk). The sample from this study was nationally representative of the English population at baseline^22^. The primary data for these analysis were collected on wave 2 of ELSA in 2004–2005 (which had an 81.5% response rate) as this was the wave in which questions about cultural engagement were first asked in the participant self-complete questionnaire. Our analyses followed up cognition 10 years later (wave 7). Ethical approval for ELSA was provided by the National Research Ethic Service, all research was performed in accordance with relevant guidelines/regulations and all participants provided informed consent.

In 2004-5, 8,780 of original participants were still alive and responded to the second wave of the ELSA survey and 4,603 of them responded to wave 7 in 2014-15. We included participants who provided full data across all variables (n = 3,449). We excluded any remaining participants who were registered blind (n = 4), providing a total sample size of 3,445. The datasets analysed during the current study are available through the UK Data Service.

### Measures

*Cultural engagement* was measured using three separate self-report scales assessing the frequency with which participants currently reported going to (i) an art gallery, museum or exhibition, (ii) the theatre, a concert or the opera, or (iii) the cinema (‘never’, ‘less than once a year’, ‘about once or twice a year’, ‘every few months’, ‘about once a month’ or ‘twice a month or more’). Due to reduced numbers in the two most frequent categories, these were collapsed together in analysis, providing a 5-point response scale.

*Cognitive measures* included memory and semantic fluency. Immediate and delayed verbal memory were assessed using a word learning task from the Health and Retirement study in which participants were presented with 10 common words by a taped voice at a rate of one word every 2 seconds. Participants then had to recall as many words as possible immediately and after a short delay (c.10 minutes) during which they complete other cognitive tests. The results from immediate and delayed recall were then summed to create an overall ‘recall’ variable. The volume of the computer was adjusted to suit the hearing needs of each participant. Participants within the same household were given different randomly assigned word lists. Semantic fluency was assessed using a task previously used in other studies including the Medical Research Council Cognitive Function and Ageing Studies (MRC CFAS) asking participants to think of as many words from a particular category (in this case animals) as possible in under 1 minute. These measures were chosen as both recall and verbal fluency have been shown to be predictors of clinically significant cognitive decline^23,24^.

*Covariates* were determined based on directed acyclic graphs (DAGs); causal diagrams used to identify variables that must be measured and controlled to obtain unconfounded effect estimates^48^. We analysed sex (with men as the reference category) and age (as a continuous variable). We indexed marital status as married or cohabiting vs other (never married, divorced, separated, widowed). Ethnicity was categorised as white vs other. Educational attainment was divided into four categories (no qualifications, GCE/O Level, A level or other higher education, and degree). Classification of occupational type across individuals’ careers was indexed using the 5-point National Statistics Socio-Economic Classification (NS-SEC). Current employment status was divided into three categories (currently working full time, currently working part time and not working). Wealth is used in ELSA analyses as a robust indicator of socioeconomic circumstances and standard of living and refers to the total net non-pension assets of an individual. In order to account or uneven distributions (in particular prominent outliers), wealth was divided into quintiles for these analyses, as follows previous precedent^49^. Self-rated health, eyesight and hearing were assessed using a 5-point Likert scale (from 1 ‘excellent’ to 5 ‘poor’). Depression was assessed using the 8-item Centre for Epidemiologic Studies Depression Scale (CES-D); a scale used extensively in a range of population cohorts^50^. Social network activity was indexed through assessing whether participants had at least monthly meetings, phone calls or emails/letters each with family members, children or friends^39^. Engagement in any civic or social activities (including political groups, neighbourhood groups, church groups, charities, evening classes or arts or music groups, social clubs, sports clubs or other societies) was assessed as a single binary variable asking about engagement over the past year.And participants were also asked about whether they currently had a hobby, used the internet or email and read a daily newspaper.

### Statistics

We used Spearman correlations to explore correlations between the three types of cultural engagement. Ordinary least squares regression was then used to estimate the effects of each of the three types of cultural engagement on each of the two cognitive outcomes separately (semantic fluency and memory). We built up our regression models gradually in order to ascertain the effect of specific categories of confounders on results. Model 1 adjusted just for baseline cognition. Model 2 additionally adjusted for demographic variables including sex, age, marital status, ethnicity, educational attainment, occupational classification, current employment status and wealth. Model 3 additionally adjusted for health-related variables including self-reported health, eyesight, hearing and depression. Model 4 additionally adjusted for activity-related variables including social network, civic engagement, whether participants had a hobby, whether participants used the internet and whether participants read a daily newspaper. All models met regression assumptions. SI Appendix Table S1 detail the different models. To manage the inevitable attrition involved in panel studies, we applied inverse probability weighting using cross-sectional weights to correct for differential nonresponse. Given that we ran an overall total of six regression models (3 exposures × 2 outcomes), a Bonferroni alpha of 0.0083 can be assumed. Applying this threshold affects only results showing associations between cinema engagement and semantic fluency (which are discussed above as inconclusive anyway), the least frequent engagement with the theatre, concert or opera, and engagement with galleries/museums in relation to semantic fluency. All other results fall below this threshold and therefore remain significant.

We carried out a series of sensitivity analyses. First, we explored baseline correlation associations between cultural engagement and cognition in order to ascertain whether any preliminary differences were evident. Second, we explored whether age was a moderating factor by rerunning analyses including age as an interaction term (cultural participation × age). Third, we stratified cognitive function at baseline using a median split and re-analysed the data. Fourth, we re-ran analyses excluding participants who reported mobility issues at baseline, including difficulty walking 100 m or more or difficulty sitting for 2 hours or more. Fifth, we re-ran analyses excluding participants who reported a dementia diagnosis at baseline or in the two years following baseline. Results of sensitivity analyses are shown in SI Appendix Tables S2–S5.

## Electronic supplementary material

Supplementary Tables
