# Supplementary material for: Cultural engagement predicts changes in cognitive function in older adults over a 10 year period: findings from the English Longitudinal Study of Ageing
Source: Sci Rep. 2018 Jul 5;8:10226. doi: 10.1038/s41598-018-28591-8 (PMC6033851; doi:10.1038/s41598-018-28591-8)
Supplement: Supplementary file 1 — Supplementary Tables [file 41598_2018_28591_MOESM1_ESM.doc]

**Supplementary Tables**

# Cultural engagement predicts changes in cognitive function in older adults over a 10 year period: findings from the English Longitudinal Study of Ageing

Daisy Fancourt PhD and Andrew Steptoe DPhil DSc

Table S1-A: Regression coefficients showing the effect of cultural engagement on cognition: model 1

|  | Memory | | | | Semantic fluency | | | |
| --- | --- | --- | --- | --- | --- | --- | --- | --- |
|  | β ± SE | t | p | CI | β ± SE | t | p | CI |
| Gallery/museum |  |  |  |  |  |  |  |  |
| Less than once a year | **0.83 ± 0.15** | **5.61** | **<.001** | **0.54-1.13** | **1.17 ± 0.28** | **4.16** | **<.001** | **0.62-1.73** |
| Once or twice a year | **1.27 ± 0.16** | **8.03** | **<.001** | **0.96-1.58** | **2.02 ± 0.30** | **6.78** | **<.001** | **1.44-2.61** |
| Every few months | **1.63 ± 0.18** | **9.24** | **<.001** | **1.28-1.98** | **3.06 ± 0.34** | **8.89** | **<.001** | **2.38-3.73** |
| Monthly or more | **1.65 ± 0.27** | **6.18** | **<.001** | **1.12-2.27** | **2.23 ± 0.51** | **4.53** | **<.001** | **1.32-3.33** |
| Theatre/concert/opera |  |  |  |  |  |  |  |  |
| Less than once a year | **1.07 ± 0.16** | **6.48** | **<.001** | **0.74-1.39** | **1.51 ± 0.31** | **4.86** | **<.001** | **0.90-2.11** |
| Once or twice a year | **0.96 ± 0.16** | **6.07** | **<.001** | **0.65-1.27** | **1.71 ± 0.30** | **5.66** | **<.001** | **1.12-2.31** |
| Every few months | **1.56 ± 0.17** | **9.26** | **<.001** | **1.23-1.89** | **2.49 ± 0.32** | **7.82** | **<.001** | **1.87-3.11** |
| Monthly or more | **1.86 ± 0.24** | **7.89** | **<.001** | **1.40-2.33** | **2.82 ± 0.43** | **6.52** | **<.001** | **1.97-3.67** |
| Cinema |  |  |  |  |  |  |  |  |
| Less than once a year | **0.81 ± 0.15** | **5.35** | **<.001** | **0.51-1.11** | **1.64 ± 0.30** | **5.46** | **<.001** | **1.05-2.23** |
| Once or twice a year | **1.16 ± 0.16** | **7.41** | **<.001** | **0.86-1.47** | **2.07 ± 0.30** | **7.01** | **<.001** | **1.49-2.65** |
| Every few months | **1.72 ± 0.17** | **9.98** | **<.001** | **1.38-2.06** | **2.47 ± 0.33** | **7.38** | **<.001** | **1.81-3.13** |
| Monthly or more | **1.45 ± 0.22** | **6.55** | **<.001** | **1.02-1.88** | **1.80 ± 0.38** | **4.71** | **<.001** | **1.05-2.55** |

REF: never. Β=beta coefficient; SE=standard error; CI=confidence intervals. N=3,445. Model adjusted for baseline cognition.

Table S1-B: Regression coefficients showing the effect of cultural engagement on cognition: model 2

|  | Memory | | | | Semantic fluency | | | |
| --- | --- | --- | --- | --- | --- | --- | --- | --- |
|  | β ± SE | t | p | CI | β ± SE | t | p | CI |
| Gallery/museum |  |  |  |  |  |  |  |  |
| Less than once a year | **0.33 ± 0.14** | **2.35** | **.019** | **0.06-0.60** | 0.43 ± 0.28 | 1.55 | .12 | -0.12-0.98 |
| Once or twice a year | **0.71 ± 0.15** | **4.67** | **<.001** | **0.41-1.00** | **1.30 ± 0.30** | **4.36** | **<.001** | **0.72-1.89** |
| Every few months | **0.86 ± 0.17** | **4.95** | **<.001** | **0.52-1.21** | **2.06 ± 0.35** | **5.89** | **<.001** | **1.37-2.74** |
| Monthly or more | **0.96 ± 0.26** | **3.74** | **<.001** | **0.45-1.46** | **1.51 ± 0.49** | **3.06** | **.002** | **0.54-2.48** |
| Theatre/concert/opera |  |  |  |  |  |  |  |  |
| Less than once a year | **0.56 ± 0.16** | **3.59** | **<.001** | **0.25-0.86** | **0.80 ± 0.31** | **2.59** | **.010** | **0.19-1.41** |
| Once or twice a year | **0.43 ± 0.15** | **2.80** | **.005** | **0.13-0.73** | **1.02 ± 0.31** | **3.32** | **.001** | **0.42-1.63** |
| Every few months | **0.86 ± 0.17** | **5.17** | **<.001** | **0.53-1.18** | **1.59 ± 0.33** | **4.88** | **<.001** | **0.95-2.23** |
| Monthly or more | **1.08 ± 0.23** | **4.65** | **<.001** | **0.62-1.53** | **1.85 ± 0.44** | **4.17** | **<.001** | **0.98-2.72** |
| Cinema |  |  |  |  |  |  |  |  |
| Less than once a year | 0.26 ± 0.14 | 1.83 | .067 | -0.02-0.54 | **0.83 ± 0.30** | **2.78** | **.005** | **0.25-1.42** |
| Once or twice a year | **0.34 ± 0.15** | **2.23** | **.026** | **0.04-0.63** | **0.93 ± 0.30** | **3.10** | **.002** | **0.34-1.51** |
| Every few months | **0.75 ± 0.17** | **4.50** | **<.001** | **0.42-1.07** | **1.07 ± 0.34** | **3.11** | **.002** | **0.39-1.74** |
| Monthly or more | **0.46 ± 0.21** | **2.16** | **.031** | **0.04-0.88** | 0.48 ± 0.39 | 1.25 | .21 | -0.27-1.24 |

REF: never. Β=beta coefficient; SE=standard error; CI=confidence intervals. N=3,445. Model adjusted for baseline cognition, sex, age, marital status, ethnicity, educational attainment, employment status, occupational classification and wealth.

**Table S1-C**: Regression coefficients showing the effect of cultural engagement on cognition: model 3

|  | Memory | | | | Semantic fluency | | | |
| --- | --- | --- | --- | --- | --- | --- | --- | --- |
|  | β ± SE | t | p | CI | β ± SE | t | p | CI |
| Gallery/museum |  |  |  |  |  |  |  |  |
| Less than once a year | 0.25 ± 0.14 | 1.81 | .07 | -0.02-0.53 | 0.35 ± 0.28 | 1.23 | .22 | -0.21-0.90 |
| Once or twice a year | **0.60 ± 0.15** | **3.93** | **<.001** | **0.30-0.90** | **1.13 ± 0.30** | **3.74** | **<.001** | **0.54-1.72** |
| Every few months | **0.77 ± 0.18** | **4.41** | **<.001** | **0.43-1.12** | **1.91 ± 0.35** | **5.37** | **<.001** | **1.21-2.60** |
| Monthly or more | **0.85 ± 0.26** | **3.32** | **<.001** | **0.35-1.36** | **1.36 ± 0.50** | **2.74** | **.006** | **0.39-2.33** |
| Theatre/concert/opera |  |  |  |  |  |  |  |  |
| Less than once a year | **0.47 ± 0.16** | **3.00** | **.003** | **0.16-0.78** | **0.67 ± 0.31** | **2.12** | **.034** | **0.05-1.28** |
| Once or twice a year | **0.33 ± 0.15** | **2.14** | **.032** | **0.03-0.63** | **0.88 ± 0.31** | **2.82** | **.005** | **0.27-1.49** |
| Every few months | **0.74 ± 0.17** | **4.47** | **<.001** | **0.42-1.07** | **1.41 ± 0.33** | **4.22** | **<.001** | **0.75-2.06** |
| Monthly or more | **0.94 ± 0.23** | **4.02** | **<.001** | **0.48-1.40** | **1.63 ± 0.45** | **3.59** | **<.001** | **0.74-2.52** |
| Cinema |  |  |  |  |  |  |  |  |
| Less than once a year | 0.21 ± 0.14 | 1.44 | .15 | -0.07-0.49 | **0.73 ± 0.30** | **2.43** | **.015** | **0.14-1.32** |
| Once or twice a year | **0.24 ± 0.15** | **1.60** | **.11** | **-0.06-0.54** | **0.76 ± 0.30** | **2.52** | **.012** | **0.17-1.36** |
| Every few months | **0.67 ± 0.17** | **4.00** | **<.001** | **0.34-0.99** | **0.94 ± 0.35** | **2.72** | **.007** | **0.26-1.62** |
| Monthly or more | 0.35 ± 0.22 | 1.64 | .10 | -0.07-0.77 | 0.33 ± 0.39 | 0.85 | .39 | -0.43-1.09 |

REF: never. Β=beta coefficient; SE=standard error; CI=confidence intervals. N=3,445. Model adjusted for baseline cognition, sex, age, marital status, ethnicity, educational attainment, employment status, occupational classification, wealth, self-reported health, eyesight, hearing and depression.

Table S1-D: Regression coefficients showing the effect of cultural engagement on cognition: model 4

|  | Memory | | | | Semantic fluency | | | |
| --- | --- | --- | --- | --- | --- | --- | --- | --- |
|  | β ± SE | t | p | CI | β ± SE | t | p | CI |
| Gallery/museum |  |  |  |  |  |  |  |  |
| Less than once a year | 0.19 ± 0.14 | 1.37 | .17 | -0.08-0.47 | 0.26 ± 0.29 | 0.90 | .37 | -0.31-0.82 |
| Once or twice a year | **0.52 ± 0.15** | **3.37** | **.001** | **0.22-0.82** | **1.02 ± 0.31** | **3.30** | **.001** | **0.41-1.62** |
| Every few months | **0.67 ± 0.18** | **3.72** | **<.001** | **0.32-1.02** | **1.75 ± 0.36** | **4.83** | **<.001** | **1.04-2.46** |
| Monthly or more | **0.74 ± 0.26** | **2.86** | **.004** | **0.23-1.25** | **1.20 ± 0.50** | **2.39** | **.017** | **0.22-2.19** |
| Theatre/concert/opera |  |  |  |  |  |  |  |  |
| Less than once a year | **0.41 ± 0.16** | **2.62** | **.009** | **0.10-0.72** | 0.58 ± 0.32 | 1.82 | .069 | -0.05-1.20 |
| Once or twice a year | 0.24 ± 0.16 | 1.53 | .13 | -0.07-0.55 | **0.74 ± 0.32** | **2.33** | **.020** | **0.12-1.37** |
| Every few months | **0.63 ± 0.17** | **3.66** | **<.001** | **0.29-0.97** | **1.23 ± 0.34** | **3.58** | **<.001** | **0.56-1.91** |
| Monthly or more | **0.80 ± 0.24** | **3.32** | **.001** | **0.33-1.27** | **1.41 ± 0.47** | **3.03** | **.002** | **0.50-2.32** |
| Cinema |  |  |  |  |  |  |  |  |
| Less than once a year | 0.16 ± 0.14 | 1.11 | 0.27 | -0.12-0.44 | **0.66 ± 0.30** | **2.18** | **.030** | **0.07-1.25** |
| Once or twice a year | 0.17 ± 0.15 | 1.13 | 0.26 | -0.13-0.48 | **0.65 ± 0.31** | **2.10** | **.036** | **0.04-1.25** |
| Every few months | **0.56 ± 0.17** | **3.31** | **0.001** | **0.23-0.89** | **0.76 ± 0.35** | **2.14** | **.032** | **0.06-1.45** |
| Monthly or more | 0.25 ± 0.22 | 1.13 | 0.26 | -0.18-0.67 | 0.14 ± 0.39 | 0.36 | .72 | -0.63-0.91 |

REF: never. Β=beta coefficient; SE=standard error; CI=confidence intervals. N=3,445. Model adjusted for baseline cognition, sex, age, marital status, ethnicity, educational attainment, employment status, occupational classification, wealth, self-reported health, eyesight, hearing, depression, social network, civic engagement, whether participants had a hobby, whether participants used the internet and whether participants read a daily newspaper.

Table S2: Regression coefficients showing baseline associations between cultural engagement and cognition

|  | Memory | | | | Semantic fluency | | | |
| --- | --- | --- | --- | --- | --- | --- | --- | --- |
|  | β ± SE | t | p | CI | β ± SE | t | p | CI |
| Gallery/museum |  |  |  |  |  |  |  |  |
| Less than once a year | **0.36 ± 0.13** | **2.77** | **.006** | **0.11-0.62** | 0.43 ± 0.27 | 1.59 | .11 | -0.10-0.96 |
| Once or twice a year | **0.40 ± 0.14** | **2.78** | **.005** | **0.12-0.69** | **0.77 ± 0.30** | **2.59** | **.010** | **0.19-1.36** |
| Every few months | **0.96 ± 0.17** | **5.60** | **<.001** | **0.62-1.29** | **1.48 ± 0.35** | **4.20** | **<.001** | **0.79-2.17** |
| Monthly or more | **0.81 ± 0.24** | **3.39** | **.001** | **0.34-1.28** | **1.81 ± 0.49** | **3.67** | **<.001** | **0.85-2.78** |
| Theatre/concert/opera |  |  |  |  |  |  |  |  |
| Less than once a year | **0.46 ± 0.15** | **3.15** | **.002** | **0.17-0.75** | 0.23 ± 0.30 | 0.75 | .45 | -0.36-0.81 |
| Once or twice a year | **0.35 ± 0.15** | **2.38** | **.017** | **0.06-0.63** | -0.09 ± 0.30 | -0.31 | .76 | -0.68-0.50 |
| Every few months | **0.47 ± 0.16** | **2.91** | **.004** | **0.15-0.79** | 0.13 ± 0.33 | 0.38 | .71 | -0.53-0.78 |
| Monthly or more | **0.53 ± 0.21** | **2.46** | **.014** | **0.11-0.95** | 0.31 ± 0.44 | 0.70 | .49 | -0.56-1.17 |
| Cinema |  |  |  |  |  |  |  |  |
| Less than once a year | 0.25 ± 0.13 | 1.92 | .055 | -0.01-0.51 | 0.21 ± 0.27 | 0.75 | .45 | -0.33-0.74 |
| Once or twice a year | 0.14 ± 0.15 | 0.96 | .34 | -0.15-0.43 | 0.28 ± 0.30 | 0.92 | .36 | -0.31-0.87 |
| Every few months | **0.55 ± 0.16** | **3.54** | **<.001** | **0.25-0.86** | **0.78 ± 0.32** | **2.42** | **.015** | **0.15-1.41** |
| Monthly or more | 0.05 ± 0.20 | 0.28 | .78 | -0.33-0.44 | 0.42 ± 0.40 | 1.04 | .30 | -0.37-1.22 |

REF: never. Β=beta coefficient; SE=standard error; CI=confidence intervals. N=3,445. Model adjusted for sex, age, marital status, ethnicity, educational attainment, employment status, occupational classification, wealth, self-reported health, eyesight, hearing, depression, social network, civic engagement, whether participants had a hobby, whether participants used the internet and whether participants read a daily newspaper.

**Table S3-A:** Regression coefficients showing the effect of cultural engagement on cognition: lower cognitive function

|  | Memory | | | | Semantic fluency | | | |
| --- | --- | --- | --- | --- | --- | --- | --- | --- |
|  | β ± SE | t | p | CI | β ± SE | t | p | CI |
| Gallery/museum |  |  |  |  |  |  |  |  |
| Less than once a year | 0.21 ± 0.18 | 1.12 | .26 | -0.15-0.57 | 0.16 ± 0.35 | 0.45 | .65 | 0.53-0.85 |
| Once or twice a year | **0.45 ± 0.21** | **2.19** | **.029** | **0.05-0.86** | **0.82 ± 0.39** | **2.08** | **.037** | 0.05-1.59 |
| Every few months | **0.68 ± 0.27** | **2.54** | **.011** | **0.15-1.20** | **1.16 ± 0.50** | **2.33** | **.020** | 0.18-2.14 |
| Monthly or more | 0.64 ± 0.37 | 1.75 | .080 | -0.08-1.37 | 0.71 ± 0.77 | 0.92 | 0.36 | -0.80-2.21 |
| Theatre/concert/opera |  |  |  |  |  |  |  |  |
| Less than once a year | **0.65 ± 0.20** | **3.18** | **.001** | **0.25-1.05** | 0.55 ± 0.40 | 1.39 | 0.17 | -0.23-1.32 |
| Once or twice a year | 0.15 ± 0.20 | 0.74 | .46 | -0.25-0.55 | **0.87 ± 0.40** | **2.21** | **.027** | **0.10-1.65** |
| Every few months | **0.88 ± 0.24** | **3.68** | **<.001** | **0.41-1.34** | 0.82 ± 0.45 | 1.80 | .072 | -0.07-1.71 |
| Monthly or more | **0.84 ± 0.32** | **2.64** | **.008** | **0.21-1.46** | 1.14 ± 0.63 | 1.79 | .073 | -0.11-2.38 |
| Cinema |  |  |  |  |  |  |  |  |
| Less than once a year | 0.06 ± 0.19 | 0.31 | .76 | -0.31-0.43 | 0.46 ± 0.36 | 1.30 | .19 | -0.24-1.16 |
| Once or twice a year | 0.14 ± 0.21 | 0.65 | .51 | -0.28-0.55 | 0.66 ± 0.41 | 1.63 | .10 | -0.13-1.46 |
| Every few months | **0.53 ± 0.24** | **2.21** | **.028** | **0.06-1.00** | 0.35 ± 0.45 | 0.77 | .44 | -0.54-1.23 |
| Monthly or more | 0.38 ± 0.29 | 1.30 | .19 | -0.19-0.94 | -0.20-0.58 | -0.33 | .74 | -1.34-0.95 |

REF: never. Β=beta coefficient; SE=standard error; CI=confidence intervals. Memory: n=1,854. Semantic fluency: n=1,752. Model adjusted for baseline cognition, sex, age, marital status, ethnicity, educational attainment, employment status, occupational classification, wealth, self-reported health, eyesight, hearing, depression, social network, civic engagement, whether participants had a hobby, whether participants used the internet and whether participants read a daily newspaper.

**Table S3-B:** Regression coefficients showing the effect of cultural engagement on cognition: higher cognitive function

|  | Memory | | | | Semantic fluency | | | |
| --- | --- | --- | --- | --- | --- | --- | --- | --- |
|  | β ± SE | t | p | CI | β ± SE | t | p | CI |
| Gallery/museum |  |  |  |  |  |  |  |  |
| Less than once a year | 0.30 ± 0.22 | 1.39 | .16 | -0.12-0.73 | 0.59 ± 0.45 | 1.29 | 0.20 | -0.30-1.48 |
| Once or twice a year | **0.70 ± 0.23** | **3.00** | **.003** | **0.24-1.16** | **1.42 ± 0.49** | **2.92** | **.004** | **0.47-2.38** |
| Every few months | **0.74 ± 0.26** | **2.88** | **.004** | **0.24-1.25** | **2.13 ± 0.54** | **3.92** | **<.001** | **1.07-3.20** |
| Monthly or more | **0.98 ± 0.36** | **2.73** | **.006** | **0.28-1.68** | 1.36 ± 0.72 | 1.89 | .059 | -0.49-2.76 |
| Theatre/concert/opera |  |  |  |  |  |  |  |  |
| Less than once a year | 0.23 ± 0.24 | 0.96 | .34 | -0.24-0.70 | 0.51 ± 0.49 | 1.05 | .30 | -0.45-1.47 |
| Once or twice a year | 0.39 ± 0.24 | 1.64 | .10 | -0.08-0.87 | 0.52 ± 0.49 | 1.06 | .29 | -0.44-1.49 |
| Every few months | 0.45 ± 0.26 | 1.77 | .077 | 0.05-0.95 | **1.43 ± 0.53** | **2.70** | **.007** | **0.39-2.46** |
| Monthly or more | **0.82 ± 0.33** | **2.46** | **.014** | **0.17-1.47** | **1.34 ± 0.66** | **2.01** | **.044** | **0.03-2.64** |
| Cinema |  |  |  |  |  |  |  |  |
| Less than once a year | 0.25 ± 0.22 | 1.14 | .25 | -0.18-0.67 | 0.82 ± 0.44 | 1.85 | .065 | -0.05-1.69 |
| Once or twice a year | 0.19 ± 0.23 | 0.83 | .41 | -0.26-0.65 | 0.63 ± 0.48 | 1.32 | .19 | -0.31-1.57 |
| Every few months | **0.64 ± 0.24** | **2.73** | **.006** | **0.18-1.10** | **1.06 ± 0.50** | **2.13** | **.034** | **0.08-2.03** |
| Monthly or more | 0.21-0.31 | 0.69 | .49 | -0.39-0.81 | 0.50 ± 0.61 | 0.82 | .41 | -0.69-1.69 |

REF: never. Β=beta coefficient; SE=standard error; CI=confidence intervals. Memory: n=1,591. Semantic fluency: n=1,693. Model adjusted for baseline cognition, sex, age, marital status, ethnicity, educational attainment, employment status, occupational classification, wealth, self-reported health, eyesight, hearing, depression, social network, civic engagement, whether participants had a hobby, whether participants used the internet and whether participants read a daily newspaper.

**Table S4-A:** Regression coefficients showing the effect of cultural engagement on cognition: excluding those with mobility issues

|  | Memory | | | | Semantic fluency | | | |
| --- | --- | --- | --- | --- | --- | --- | --- | --- |
|  | β ± SE | t | p | CI | β ± SE | t | p | CI |
| Gallery/museum |  |  |  |  |  |  |  |  |
| Less than once a year | 0.29 ± 0.15 | 1.90 | .058 | -0.01-0.59 | 0.23 ± 0.31 | 0.74 | 0.46 | -0.38-0.83 |
| Once or twice a year | **0.62 ± 0.17** | **3.71** | **<.001** | **0.29-0.94** | **1.04 ± 0.34** | **3.09** | **.002** | **0.38-1.69** |
| Every few months | **0.73 ± 0.20** | **3.73** | **<.001** | **0.35-1.11** | **1.72 ± 0.39** | **4.36** | **<.001** | **0.94-2.49** |
| Monthly or more | **0.88 ± 0.27** | **3.27** | **.001** | **0.35-1.41** | **1.14 ± 0.55** | **2.09** | **.037** | **0.07-2.21** |
| Theatre/concert/opera |  |  |  |  |  |  |  |  |
| Less than once a year | **0.47 ± 0.17** | **2.75** | **.006** | **0.13-0.80** | **0.78 ± 0.34** | **2.28** | **.023** | **0.11-1.46** |
| Once or twice a year | **0.37 ± 0.17** | **2.18** | **.029** | **0.04-0.70** | **0.82 ± 0.34** | **2.40** | **.016** | **0.15-1.49** |
| Every few months | **0.82 ± 0.19** | **4.42** | **<.001** | **0.46-1.18** | **1.37 ± 0.38** | **3.66** | **<.001** | **0.64-2.11** |
| Monthly or more | **0.91 ± 0.24** | **3.83** | **<.001** | **0.45-1.38** | **1.44 ± 0.48** | **3.00** | **.003** | **0.50-2.39** |
| Cinema |  |  |  |  |  |  |  |  |
| Less than once a year | 0.14 ± 0.15 | 0.92 | .36 | -0.16-0.44 | **0.66 ± 0.31** | **2.14** | **.033** | **0.06-1.27** |
| Once or twice a year | 0.32 ± 0.17 | 1.93 | .054 | 0.01-0.65 | **0.83 ± 0.34** | **2.46** | **.014** | **0.17-1.49** |
| Every few months | **0.64 ± 0.18** | **3.63** | **<.001** | **0.30-0.99** | **0.92 ± 0.36** | **2.57** | **.010** | **0.22-1.62** |
| Monthly or more | 0.33 ± 0.22 | 1.53 | .13 | -0.09-0.76 | 0.30 ± 0.44 | 0.69 | .49 | -0.56-1.17 |

REF: never. Β=beta coefficient; SE=standard error; CI=confidence intervals. N=2,908. Model adjusted for baseline cognition, sex, age, marital status, ethnicity, educational attainment, employment status, occupational classification, wealth, self-reported health, eyesight, hearing, depression, social network, civic engagement, whether participants had a hobby, whether participants used the internet and whether participants read a daily newspaper.

**Table S5-A:** Regression coefficients showing the effect of cultural engagement on cognition: excluding those with dementia diagnosis at baseline or in the 2 years following baseline

|  | Memory | | | | Semantic fluency | | | |
| --- | --- | --- | --- | --- | --- | --- | --- | --- |
|  | β ± SE | t | p | CI | β ± SE | t | p | CI |
| Gallery/museum |  |  |  |  |  |  |  |  |
| Less than once a year | **0.29 ± 0.14** | **2.01** | **.045** | **0.01-0.57** | 0.29 ± 0.28 | 1.01 | .31 | -0.27-0.85 |
| Once or twice a year | **0.62 ± 0.16** | **3.92** | **<.001** | **0.31-0.92** | **1.11 ± 0.31** | **3.55** | **<.001** | **0.50-1.73** |
| Every few months | **0.73 ± 0.19** | **3.95** | **<.001** | **0.37-1.10** | **1.70 ± 0.37** | **4.60** | **<.001** | **0.98-2.43** |
| Monthly or more | **0.88 ± 0.26** | **3.42** | **.001** | **0.38-1.39** | **1.07 ± 0.51** | **2.07** | **.038** | **0.06-2.08** |
| Theatre/concert/opera |  |  |  |  |  |  |  |  |
| Less than once a year | **0.46 ± 0.16** | **2.90** | **.004** | **0.15-0.76** | 0.52 ± 0.31 | 1.64 | .10 | -0.10-1.13 |
| Once or twice a year | 0.28 ± 0.16 | 1.77 | .077 | -0.03-0.59 | **0.75 ± 0.31** | **2.39** | **.017** | **0.13-1.37** |
| Every few months | **0.66 ± 0.18** | **3.77** | **<.001** | **0.32-1.00** | **1.16 ± 0.35** | **3.31** | **.001** | **0.47-1.84** |
| Monthly or more | **0.85 ± 0.23** | **3.70** | **<.001** | **0.40-1.30** | **1.32 ± 0.46** | **2.87** | **.004** | **0.42-2.22** |
| Cinema |  |  |  |  |  |  |  |  |
| Less than once a year | 0.14 ± 0.14 | 1.00 | .32 | -0.14-0.42 | **0.62 ± 0.29** | **2.19** | **.029** | **0.06-1.18** |
| Once or twice a year | 0.15 ± 0.16 | 0.97 | .33 | -0.16-0.46 | **0.62 ± 0.31** | **1.96** | **.050** | **0.0002-1.23** |
| Every few months | **0.55 ± 0.17** | **3.28** | **.001** | **0.22-0.88** | **0.69 ± 0.34** | **2.04** | **.041** | **0.03-1.35** |
| Monthly or more | 0.32 ± 0.21 | 1.50 | .13 | -0.10-0.73 | 0.13 ± 0.42 | 0.30 | .76 | -0.70-0.96 |

REF: never. Β=beta coefficient; SE=standard error; CI=confidence intervals. N=3,314. Model adjusted for baseline cognition, sex, age, marital status, ethnicity, educational attainment, employment status, occupational classification, wealth, self-reported health, eyesight, hearing, depression, social network, civic engagement, whether participants had a hobby, whether participants used the internet and whether participants read a daily newspaper.
